# Stabilities of bisphenol A diglycidyl ether, bisphenol F diglycidyl ether, and their derivatives under controlled conditions analyzed using liquid chromatography coupled with tandem mass spectrometry

**DOI:** 10.1007/s00216-019-02016-5

**Published:** 2019-07-19

**Authors:** Natalia Szczepańska, Paweł Kubica, Błażej Kudłak, Jacek Namieśnik, Andrzej Wasik

**Affiliations:** Department of Analytical Chemistry, Faculty of Chemistry, Gdansk University of Technology, 11/12 Narutowicza Str., 80-233 Gdańsk, Poland

**Keywords:** BADGE stability, BADGE and BFDGE derivatives, Liquid chromatography–tandem mass spectrometry

## Abstract

Bisphenol A diglycidyl ether (BADGE), bisphenol F diglycydyl ether (BFDGE), and their related compounds are widely used as precursors in production of epoxy resins. The high reactivity of these compounds makes the development of analytical methodologies that ensure appropriate metrological accuracy crucial. Consequently, we aimed to determine whether and to what extent the composition of the solution and storage conditions affect the stability of selected BADGE and BFDGE derivatives. The stabilities of these compounds were studied using liquid chromatography–tandem mass spectrometry with electrospray ionization (HPLC-ESI–MS/MS). The chromatographic method elaborated here has allowed for separation of the analytes in time shorter than 6 min, for both methanol and acetonitrile-based mobile phases. The obtained calibration curves for all analytes were linear in the range tested. The values of limit of detection (LODs) were in the range of 0.91–2.7 ng/mL, while values of limit of quantitation (LOQs) were in the range of 2.7–5.7 ng/mL. The chosen experimental conditions were compared in terms of the content of organic solvent in solution, storage temperature, and time. Our results show that the content of BADGE, BADGE·HCl, BFDGE, three-ring NOGE decreased with increasing water content (> 40% *v*/*v*). For BADGE and three-ring NOGE, significant changes in concentration were noted as early as 24 h after the test solutions had been prepared. In addition, a reduction in the storage temperature (4 to − 20 °C) reduced the rate of transformation of the monitored analytes. Our study will increase quality control in future research and may increase the reliability of the obtained results.

**Electronic supplementary material:**

The online version of this article (10.1007/s00216-019-02016-5) contains supplementary material, which is available to authorized users.

**Figure:**
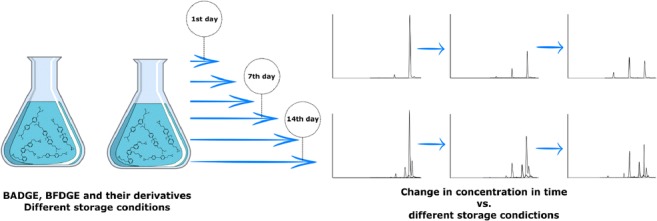
Graphical abstract

## Introduction

Undoubtedly, plastics are one of the most versatile and multifunctional materials; they are used in all fields of technology, both at home and in industry. Last year, it was reported that world’s production of plastic had reached ca. 348 million tons [1, 2]. Despite the many benefits of their use, the negative effects of plastics on the natural environment, as well as the health of living organisms, are increasingly being reported [3–5]. One xenobiotic that is a potential toxin is bisphenol A (BPA). This compound is believed to have estrogenic activity and be toxic and genotoxic [6, 7]. Unfortunately, in addition to BPA, many other biologically active compounds are used in the production of polymeric materials [8], including bisphenol A diglycidyl ether (BADGE), a synthetic compound obtained from a condensation reaction between epichlorohydrin and BPA [9]. Similarly, bisphenol F diglycidyl ether (BFDGE) is produced in the reaction between phenol–formaldehyde resins (novolac) and epichlorohydrin [1]. Furthermore, commercial packaging materials may act as sources of materials of unknown character; thus, their identification using rapid instrumental methods is necessary [10]. Other analogs (e.g., novolac glycidyl ethers (NOGE)), which contain three to eight rings, are also used commercially [9]. These compounds are mainly used for the production of epoxy resins, such as (i) interior coatings for food packaging, (ii) components of powder coatings, and (iii) components of dental resins [11–13]. Numerous studies have demonstrated that external factors may cause the release of these substances from the parent material, and the released compounds can subsequently undergo transformation into derivatives [14]. In 1991, Begley et al. confirmed the migration of a diglycidyl ether of BPA from microwave susceptor packaging into liquid simulated food [15]. Hydrolyzed derivatives, such as BADGE·2H_2_O, BADGE·H_2_O, BFDGE·2H_2_O, and BFDGE·H_2_O, can be created during storage when food coatings come into contact with aqueous and acidic foodstuffs. Chlorinated derivatives, on the other hand, may be generated during the thermal coating treatment [14, 16]. In Table 1, key information about the BADGE and BFDGE derivatives is summarized.

**Table 1:**
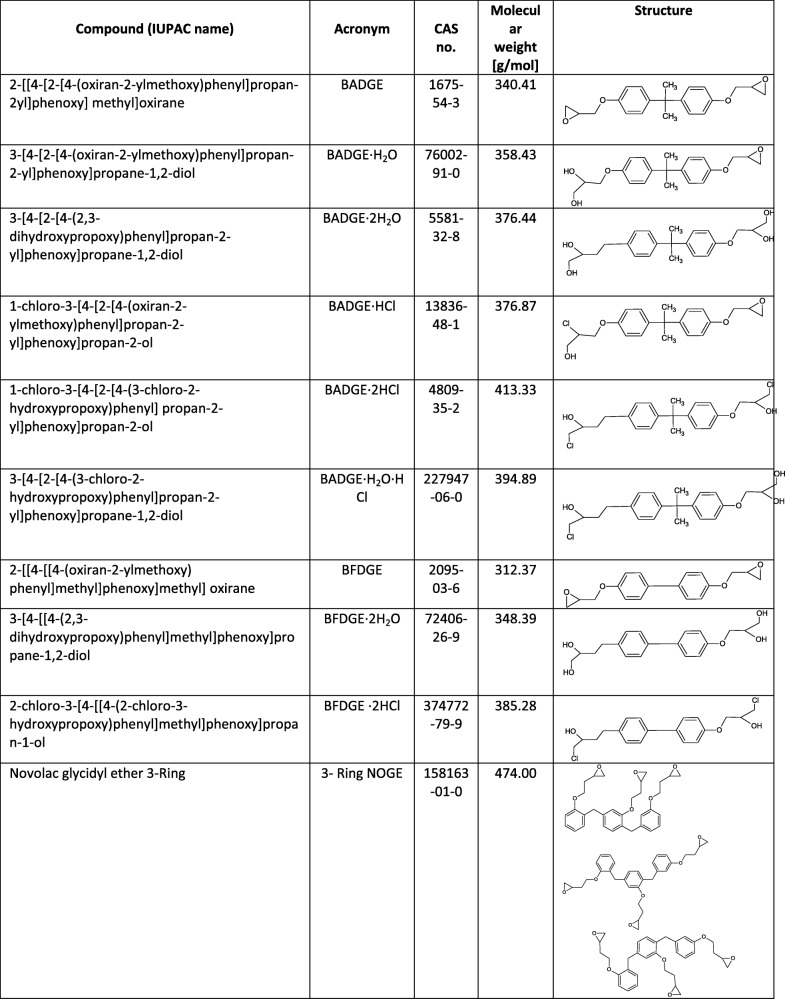
Key information about BADGE, BFDGE, and their derivatives

The compounds released from the material surface and their transformation products may enter organisms or the environment. Like BPA, these compounds can act as endocrine disruptors. BADGE and their transformation products have proven estrogenic and androgen antagonist activities [14, 17]. Moreover, in vitro assays have indicated that these chemicals have both genotoxic and cytotoxic effects, as well as developmental and reproductive toxicity [9, 18]. Similarly, for BFDGE, cytotoxic, genotoxic, and mutagenic activities have been reported. Moreover, BFDGE shows cytotoxic effects on human colorectal adenocarcinoma cell lines [18, 19]. In 2006, the European Food Safety Agency completed its risk assessment of BPA in food contact materials, and, in 2011, the use of BPA in polycarbonate feeding bottles for infants was prohibited in the European Union (EU) (European Commission Directive 2011/8/EU). Considering the high biological activity of these compounds, the development of appropriate analytical procedures to distinguish and monitor concentrations of BADGE and BFDGE analogs in various sample media is urgently required. A review of the literature reveals that current efforts have focused primarily on the identification and quantitative determination of BADGE derivatives released from the surfaces of packaging materials [14, 20, 21]. However, there is significantly less information about the content of these compounds in biological samples. Nevertheless, the presence of BADGE and BFDGE derivatives has been confirmed in plasma [18], urine [22], adipose tissue [23], the air [24], water [25], dust [26], and soil samples [9]. Considering the high reactivity of BADGE and BFDGE analogs triggered by external factors [9], appropriate storage conditions, solvents for sample preparation, and storage time between analyses should be defined and used in future studies of these compounds. Thus, the main objective of this study was to determine the stability of the BADGE and BFDGE derivatives under selected storage conditions, after different storage times, and in the presence of different organic solvent contents. Although there is a great deal of information concerning the methodologies used to identify and quantify these compounds, as well as the procedures for ensuring high quality results, there is little information about how storage conditions and solution preparation affect the stability of the analytes. This information is particularly important for sample and solution preparation, as well as for ensuring the performance of quantitative analyses. We believe that our results represent a valuable source of information on stability of BADGE, BFDGE, and their derivatives and will help to improve the quality of analytical results by better understanding the transformation products.

## Materials and methods

### Chemicals

All standards used in the study were obtained from Sigma-Aldrich (St. Louis, USA): bisphenol A diglycidyl ether (BADGE, CAS no. 1675-54-3), bisphenol A (3-chloro-2-hydroxypropyl)(2,3-dihydroxypropyl) ether (BADGE·HCl·H_2_O, CAS no. 227947-06-0), bisphenol A (2,3-dihydroxypropyl) glycidyl ether (BADGE·H_2_O, CAS no. 76002-91-0), bisphenol A (3-chloro-2-hydroxypropyl) glycidyl ether (BADGE·HCl, CAS no. 13836-48-1), bisphenol A bis(2,3-dihydroxypropyl) ether (BADGE·2H_2_O, CAS no. 5581-32-8), bisphenol A bis(3-chloro-2-hydroxypropyl) ether (BADGE·2HCl, CAS no. 4809-35-2), bisphenol F diglicydyl ether (mixture of isomers) (BFDGE, CAS no. 2095-03-6), bisphenol F bis(2,3-dihydroxypropyl) ether (BFDGE·H_2_O, CAS no. 72406-26-9), bisphenol F bis(3-chloro-2-hydroxypropyl) ether (BFDGE·2HCl, CAS no. 374772-79-9), and three-ring novolac glycidyl ether (mixture of isomers) (CAS no. 158163-01-0). The internal standard (IS) d_10_-labeled BADGE (CAS no. 1675-54-3) was supplied by Cambridge Isotope Laboratories Inc. (Cambridge, UK). Methanol (MeOH) (CAS no. 67-56-1) and acetonitrile (ACN, CAS no. 75-05-8) used during the sample preparation procedure and as mobile phase components were of liquid chromatography–mass spectrometry (LC-MS) hypergrade purity and were obtained from Merck KGaA (Darmstadt, Germany). Ammonium formate (CAS no. 540-69-2) was purchased from Sigma-Aldrich (St. Louis, USA). Ultrapure water was produced by a Milli-Q Gradient A10 system equipped with an EDS-Pak cartridge to remove endocrine disrupting compounds (Merck–Millipore, Germany).

### Preparation of standards and calibration solutions

Individual stock solutions (0.5 mg/mL) of all analytes were prepared separately by dissolving accurately weighted amounts of analytical standards in MeOH. The working solution was obtained by mixing the stock solutions, followed by dilution with MeOH. All solutions were stored at − 80 °C. The calibration solutions were prepared by diluting the working solution with MeOH to obtain a six-point calibration curve (5.0, 25.0, 50.0, 75.0, 100.0, and 150.0 ng/mL). A stock solution of the IS was prepared at a concentration of 2.5 μg/mL for use in all analyses. In each calibration solution, the concentration of IS was 50 ng/mL. Fresh calibration solutions were prepared for each sample batch.

### Preparation of model solutions

Eighteen model solutions were prepared by diluting the working solution with MeOH or with a MeOH/H_2_O mixture (10.0 mL each). The concentration of each analyte in the prepared model solution was 100 ng/mL. To verify how the content of organic solvent affected the stability of the compounds, solutions were prepared with different MeOH contents: 100%, 80%, 60%, 40%, 20%, and 0.1% (in triplicate). Each solution was stored at different temperatures (20, 4, and − 20 °C) to determine the effect of the temperature on the model solutions in terms of the stability of the compounds. Chromatographic analyses were carried out immediately after the preparation of the solutions (*t*_0_), as well as after 1, 2, 3, 4, 7, and 14 days (denoted *t*_1_, *t*_2_, *t*_3_, *t*_4_, *t*_7_, and *t*_14_, respectively). Before each analysis, IS was added to each individual sample. After sampling, each solution was immediately returned to storage without access to the light.

### MS/MS conditions

All analyses were performed using an LC-MS-8060 triple quadrupole mass spectrometer (Shimadzu, Japan) equipped with an electrospray ionization source (ESI) working in positive multiple reaction mode (MRM). The parameters of the ion source were set as in Aszyk et al. [27], while the optimization of MRM conditions was performed via an infusion of a 100 ng/mL solution of each substance by flow injection analysis (FIA). The MRM transitions were monitored only for specific detection time (± 1 min of *t*_R_ of analyte) to increase sensitivity. Data acquisition and quantification were accomplished using the LabSolutions v5.85 software. The optimum detection conditions are presented in Table 2.

**Table 2:** Parameters of the monitored ion transitions

Compound	Molecular formula	Precursor ion ➢ quantitation ➢ confirmation M+NH_4_^+^ (*m*/*z*)	Collision energy [V]
BFDGE·2H_2_O	C_19_H_24_O_6_	366.00	
		➢ 181.15 ➢ 107.15	− 16 − 28
BADGE·2H_2_O	C_21_H_28_O_6_	394.00	
		➢ 209.10 ➢ 135.10	− 16 − 31
BADGE·H_2_O	C_21_H_26_O_5_	376.00	
		➢ 209.20 ➢ 191.25	− 14 − 20
BFDGE	C_19_H_20_O_4_	329.90	
		➢ 163.10 ➢ 133.20	− 13 − 16
BADGE·H_2_O·HCl	C_21_H_27_ClO_5_	412.00	
		➢ 227.10 ➢ 135.23	− 17 − 33
BFDGE·2HCl	C_19_H_22_Cl_2_O_4_	402.00	
		➢ 181.00 ➢ 199.10	− 22 − 13
BADGE	C_21_H_24_O_4_	358.00	
		➢ 191.15 ➢ 135.25	− 15 − 28
BADGE·HCl	C_21_H_25_ClO_4_	394.00	
		➢ 227.10 ➢ 135.20	− 14 − 30
3-ring·NOGE	C_29_H_30_O_6_	492.20	
		➢ 163.20 ➢ 107.15	− 21 − 40
BADGE·2HCl	C_21_H_26_Cl_2_O_4_	430.00	
		➢ 107.10 ➢ 227.20	− 51 − 17

### High-performance liquid chromatography (HPLC) conditions

Chromatographic separation was carried out using the ultra-performance liquid chromatography (UPLC) Nexera X2 system (Shimadzu, Japan), which consisted of a DGU-20A5R degasser, CBM-20A controller, LC-30AD binary pump, SIL-30AC autosampler, and CTO-20AC column oven. The separation was achieved using Kinetex® XB-C8 column (100 × 2.1 mm, 2.6 μm in core-shell technology). The column oven temperature was set to 45 °C, the flow rate was kept at 1.0 mL/min, and the injection volume was set to 2.0 μL. The mobile phase used for the separation was 40 mM ammonium formate (component A) and MeOH (component B). The chromatographic separation was performed in gradient elution mode: 0 min (35% B), 7 min (85% B), and 9 min (85% B). After each analysis, the initial column conditions were restored over 5 min.

## Results and discussion

### Separation and detection of BADGE and BFDGE derivatives

**Fig. 1:**
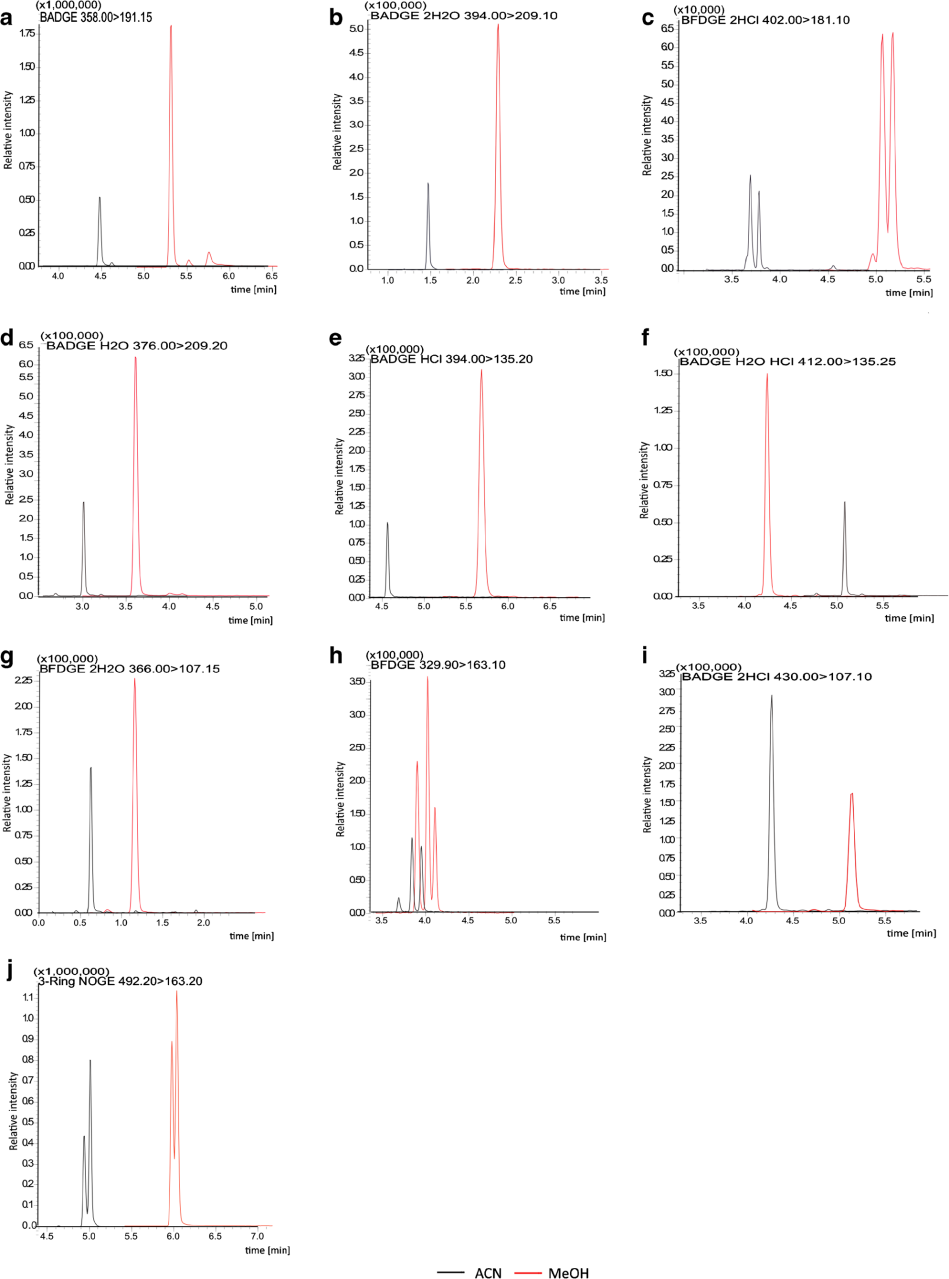
**a**–**j** Chromatograms obtained using ACN and MeOH as the organic components of the mobile phase

In this study, a rapid LC–tandem MS (MS/MS) method was developed for the analysis of BADGE, BFDGE, and their derivatives. Since these compounds tend to form adducts in positive mode, i.e., [M+NH_4_]^+^, [M+Na]^+^, and [M+K]^+^ [14, 28], ammonium acetate and ammonium formate buffer (5.0, 10.0, 25.0, 40.0, and 50.0 mM) were tested as the aqueous components of the mobile phase. Ammonium acetate buffer was eliminated in the preliminary studies because it resulted in significant signal suppression (data not shown). On the basis of the obtained spectra, ammonium formate enhances the response, and thus, the largest peak areas for the most of target compounds were obtained. Finally, 40.0 mM ammonium formate buffer was chosen for further studies because of the symmetric peak shape (tailing factor value increase from 0.95 to 1.10) and sensitivity, which allowed us to obtain a low limit of detection (LOD) values. In preliminary studies, MeOH and ACN were tested as the main organic components of the mobile phase. However, in the case of ACN, smaller response for all analytes was noted. A comparison of the influence of the organic component on the peak shapes and intensity is presented in Fig. 1.

**Fig. 2:**
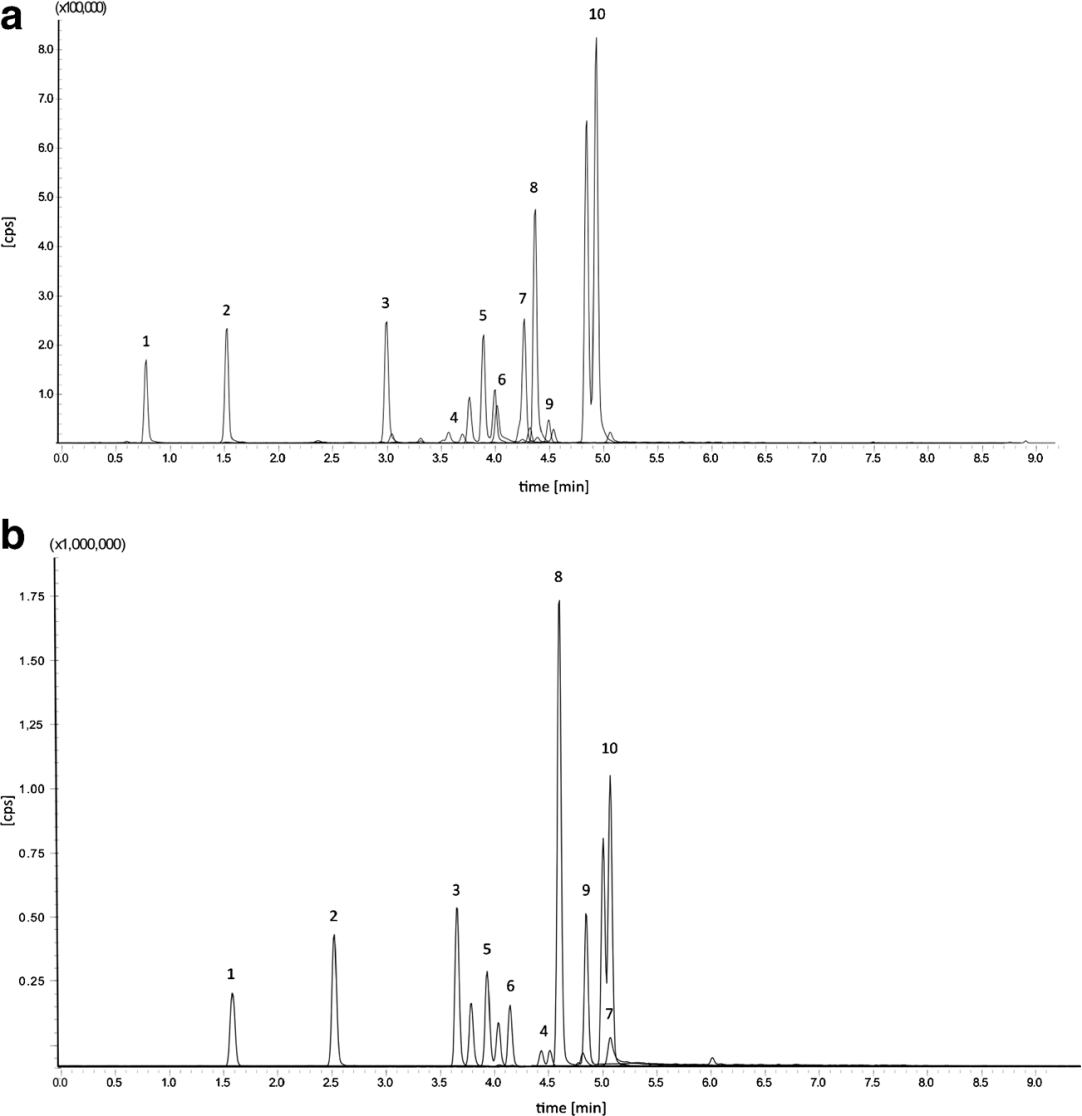
Chromatograms obtained with Kinetex ® XB-C8 column. **a** Chromatogram of mixture of analytes: (1) BFDGE·2H_2_O, (2) BADGE·2H_2_O, (3) BADGE·H_2_O, (4) BFDGE, (5) BADGE·H_2_O·HCl, (6) BFDGE·2HCl, (7) BADGE·2HCl, (8) BADGE, (9) BADGE·HCl, and (10) three-ring NOGE using ACN as a component of mobile phase. **b** Final chromatogram obtained using MeOH as a component of mobile phase

When MeOH was used as the mobile phase, the intensities of most peaks were two to three times higher than those when ACN was used. The biggest difference can be observed for the MRM transition of BADGE·H_2_O·HCl, where the response obtained using MeOH is about 20 times higher than that obtained using ACN. This phenomenon has already been reported for this family of compounds [14, 16]. Moreover, the use of MeOH resulted in better peak shapes than those obtained using ACN. Chromatograms obtained using ACN and MeOH as the organic components in the mobile phase are presented in Fig. 2.

The gradient program was optimized to obtain a good separation of the analytes and increase the retention time repeatability. As a result, the separation of the isomers of BFDGE, BFDGE·2HCl, and three-ring NOGE was possible in 5 min (cf. Fig. 2c).

### Method validation

The linear calibration equations were obtained from 6-point calibration curves (5, 25, 50, 75, 100, and 150 ng/mL), that were made by plotting the ratios of analyte peak area to IS peak area versus corresponding concentrations. The weigh factor 1/*x* was applied to all calibration curves equations to ensure increased accuracy in the lower levels of concentrations. Calibration curves were linear in the tested concentration range from 5 to 150 ng/mL and the correlation coefficients were found to be greater than 0.9984 for all the compounds. The values of limit of detection (LOD) were calculated according to the formula: LOD = 3.3 × *S*_b_/*a*, where *S*_b_ is the standard deviation of the intercept of the calibration curve, and *a* is the slope of the calibration curve. The values of limit of quantitation (LOQ) were defined as 3 × LOD. The LOQ values were in the range from 2.7–5.7 ng/mL. The values of calibration parameters are presented in Table 3. Obtained values are similar to those reported in literature by other authors [22, 29, 30].

**Table 3:** Weighed (1/*x*) calibration curve equations, standard deviations, LOD, and LOQ values obtained during method validation

Analyte	Cal. curve equation *y* = *ax* + *b*	*S*_a_	*S*_b_	*r*	LOD (ng/mL)	LOQ (ng/mL)
BADGE	*y* = 0.03047*x* − 0.015	0.00047	0.017	0.9988	1.9	5.7
BADGE·H_2_O	*y* = 0.01208*x* − 0.0100	0.00019	0.0069	0.9989	1.9	5.7
BADGE·2H_2_O	*y* = 0.00944*x* − 0.0077	0.00010	0.0039	0.9984	1.3	4.0
BADGE·HCl	*y* = 0.005091*x* − 0.0006	0.000037	0.0014	0.9992	0.91	2.7
BADGE·2HCl	*y* = 0.01141*x* − 0.0219	0.00025	0.0093	0.9952	2.7	8.1
BADGE·H_2_O·HCl	*y* = 0.004189*x* − 0.0018	0.000043	0.0016	0.9990	1.3	3.8
BFDGE	*y* = 0.006371*x* − 0.0042	0.000090	0.0033	0.9993	1.7	5.2
BFDGE·2H_2_O	*y* = 0.006056*x* − 0.0037	0.000074	0.0030	0.9979	1.6	4.9
BFDGE·2HCl	*y* = 0.001146*x* − 0.00013	0.000015	0.00054	0.9992	1.6	4.7
3-ring NOGE	*y* = 0.01790*x* − 0.0044	0.00025	0.0095	0.9986	1.8	5.3

*S*_*a*_ standard deviation of the slope of calibration curve, *S*_*b*_ standard deviation of intercept of calibration curve

### Analyte stability

**Fig. 3:**
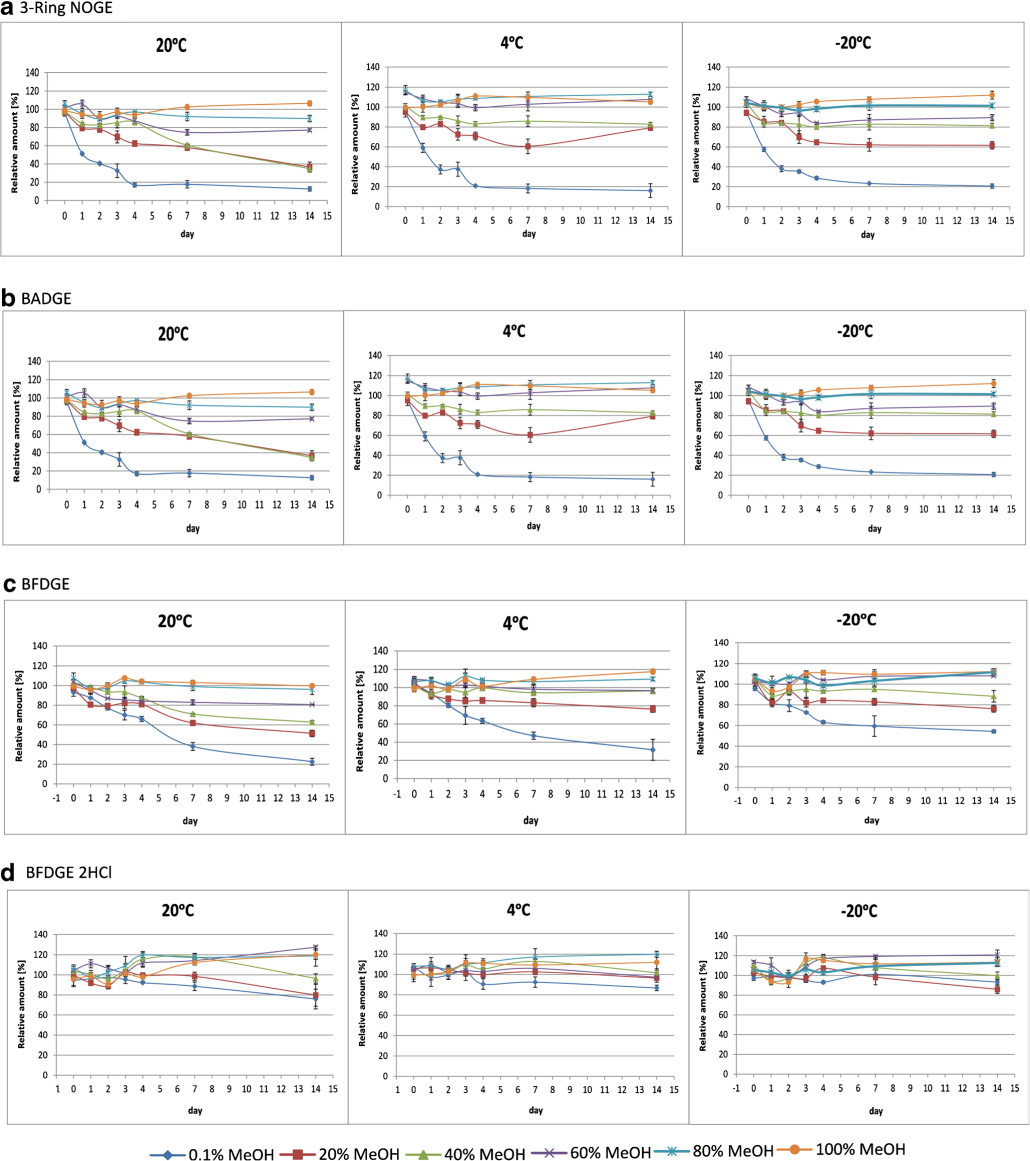
Effects of the time and temperature (20°C, 4°C and -20°C) on the stability of the tested compounds: a) 3-Ring NOGE, b) BADGE, c) BFDGE, d) BFDGE · 2HCl

Our main objective was to evaluate the stability of the BADGE and BFDGE derivatives at different temperatures (20, 4, and − 20 °C) as well as the organic solvent content (100%, 80%, 60%, 40%, 20%, and 0.1% MeOH). The samples were analyzed together with freshly made calibration solutions (for the preparation of calibration curves) before the analysis of each set of analytes. The stability was evaluated by comparing determined concentrations of the stored samples with freshly made samples. In Fig. 3, the effects of time and storage temperature on the concentration of monitored compounds are shown.

The data are presented as the average of three measurements for each sample. The repeatability of the results was expressed as the coefficient of variation (CV). For all samples, the CV values were smaller than 8%. In most cases (BADGE·2H_2_O, BADGE·H_2_O, BFDGE, BADGE·H_2_O·HCl, BADGE, BADGE·2HCl, BADGE·HCl, and three-ring NOGE), the concentration of the compounds decreased with increasing water content in the solution. In the case of higher methanol content (above 80%), the concentration of the analyte remained close to the initial value regardless of the storage time. The most important changes in concentration were registered in the case of three-ring NOGE. Regardless of the storage temperature of the solutions, 24 h after the beginning of the experiment, there was an almost 50% reduction in the initial concentration of the analyte for the samples with the highest water content. The data (cf. Fig. 3a) indicate that a higher water content in solution resulted in a more significant difference in the concentrations of the tested compounds with time. In the case of the samples containing more than 60% MeOH, the average decrease reached 10–25%. After 4 days, the analyte concentrations at room temperature reached 12% of the initial concentration, and further significant changes during the following days were not noticeable. A similar trend was observed for BFDGE and BADGE (cf. Fig. 3a, b). However, the concentration drop was less severe. After the first 24 h of the experiment, the concentrations of these compounds in the samples stored at room temperature (MeOH contents from 0.1 to 60.0%) had decreased by approximately 10–20% compared to the samples with 80% MeOH (where no significant drop was recorded).

After 7 days, the concentrations of BFDGE and BADGE in the 60% MeOH solution stored at room temperature dropped to ca. 30%, whereas, in the case of the solution with a MeOH content of 0.1%, the values had decreased by 65%. After 14 days, for BFDGE and BADGE, the decrease in concentration reached, respectively, 35% and 80% with respect to the initial values. The same trend was observed for the samples stored at 4 and − 20 °C. However, the lower the storage temperature of the samples, the lower the decrease in the concentrations of all analytes. A decrease in the BFDGE·2HCl concentration (down to 80–90% of the initial concentration value) (cf. Fig. 3d) was observed for sample solutions containing less than 40% MeOH. Based on data obtained for BFDGE·2H_2_O, BADGE·H_2_O, BADGE·2H_2_O, and BADGE·H_2_O·HCl (cf. Electronic Supplementary Material (ESM) Fig. S1, we concluded that the compounds were stable for the first 4 days of the experiment, regardless of the water content in solutions and temperature. Similar results were obtained by Chang et al., who found that BADGE hydroxy derivatives are stable in water for 5 days [8]. We found that, after a certain period of time (> 4 days), the concentration of some compounds increased (namely BADGE·2H_2_O, BADGE·H_2_O, BADGE·H_2_O·HCl, BADGE·2HCl, and BFDGE·2H_2_O). For most of the compounds, an increase in concentration by ca. 20–25% was noted. The only exception was BADGE·2H_2_O stored at room temperature in a 0.1% MeOH solution. The increase in concentration compared to the initial value was as high as 60% (cf. ESM Fig. S1c).

The concentrations of compounds in solutions with high water contents (< 20%) may change because the analytes were transformed into derivative compounds. This phenomenon may be explained by the presence of highly reactive epoxide groups in BADGE and BFDGE. The presence of a high concentration of water in these samples could result in the transformation of the epoxides into hydroxy and chloro derivatives. A high water content (60.0–99.9%) in solutions is suspected to have positive impact on such reactions. The obtained results led us to the conclusion that BADGE and BFDGE, as well as BADGE·H_2_O, were transformed into BADGE·H_2_O, BADGE·2H_2_O, BADGE·H_2_O·HCl, and BFDGE·2H_2_O, and numerous reports of these transformations have been published [30–33]. The obtained results indicate that the formation of derivatives not only occurs in contact with the packaging during processing but also in the laboratory under controlled conditions.

Furthermore, we investigated whether the decrease in the concentration of the monitored analytes was caused by their deposition on the vial surface. After 14 days, the remaining solution was removed from the vials, and they were dried in a gentle stream of N_2_. Then, a fresh 10-mL portion of MeOH containing the IS (50 ng/mL) was added to each vial. Next, the solutions were shaken vigorously for 5 min. Subsequently, the samples were immediately transferred to 2-mL glass vials and analyzed. In the solutions containing 0.1% and 20% MeOH, four analytes (three-ring NOGE, BADGE·HCl, BADGE·H_2_O·HCl, and BFDGE·2HCl) were detected. The obtained results are summarized in Table 4.

**Table 4:** Concentration of analytes (ng/mL) in samples after rinsing vials with 10 mL MeOH

Sample		3-ring NOGE	BADGE·HCl	BADGE·H_2_O·HCl	BFDGE·2HCl
20 °C	0.1% MeOH	33.74 ± 0.34	38.35 ± 0.24	3.10 ± 0.46	5.75 ± 0.28
	20% MeOH	26.25 ± 0.93	15.25 ± 0.09	ND	ND
4 °C	0.1% MeOH	16.45 ± 0.27	23.87 ± 0.71	2.98 ± 0.07	3.26 ± 0.55
	20% MeOH	6.97 ± 0.19	10.34 ± 0.11	ND	ND
− 20 °C	0.1% MeOH	12.55 ± 0.06	22.92 ± 0.08	2.85 ± 0.18	1.25 ± 0.11
	20% MeOH	4.01 ± 0.15	9.80 ± 0.13	ND	ND

*ND* not detected

The data in Table 4 suggest that the significant reduction in the concentrations of three-ring NOGE and BADGE·HCl may be due to the deposition of these compounds on the walls of the vial. The concentration decrease may also result from the transformation of the compounds into derivatives, although to a lesser extent. In addition, the presence of BADGE·H_2_O·HCl and BFDGE·2HCl in the solutions was observed. However, the amounts detected were insignificant; thus, the decrease in concentration is likely to result from the formation of derivatives.

### Repeatability of transformation by analysis of single sets of chosen compounds

**Fig. 4:**
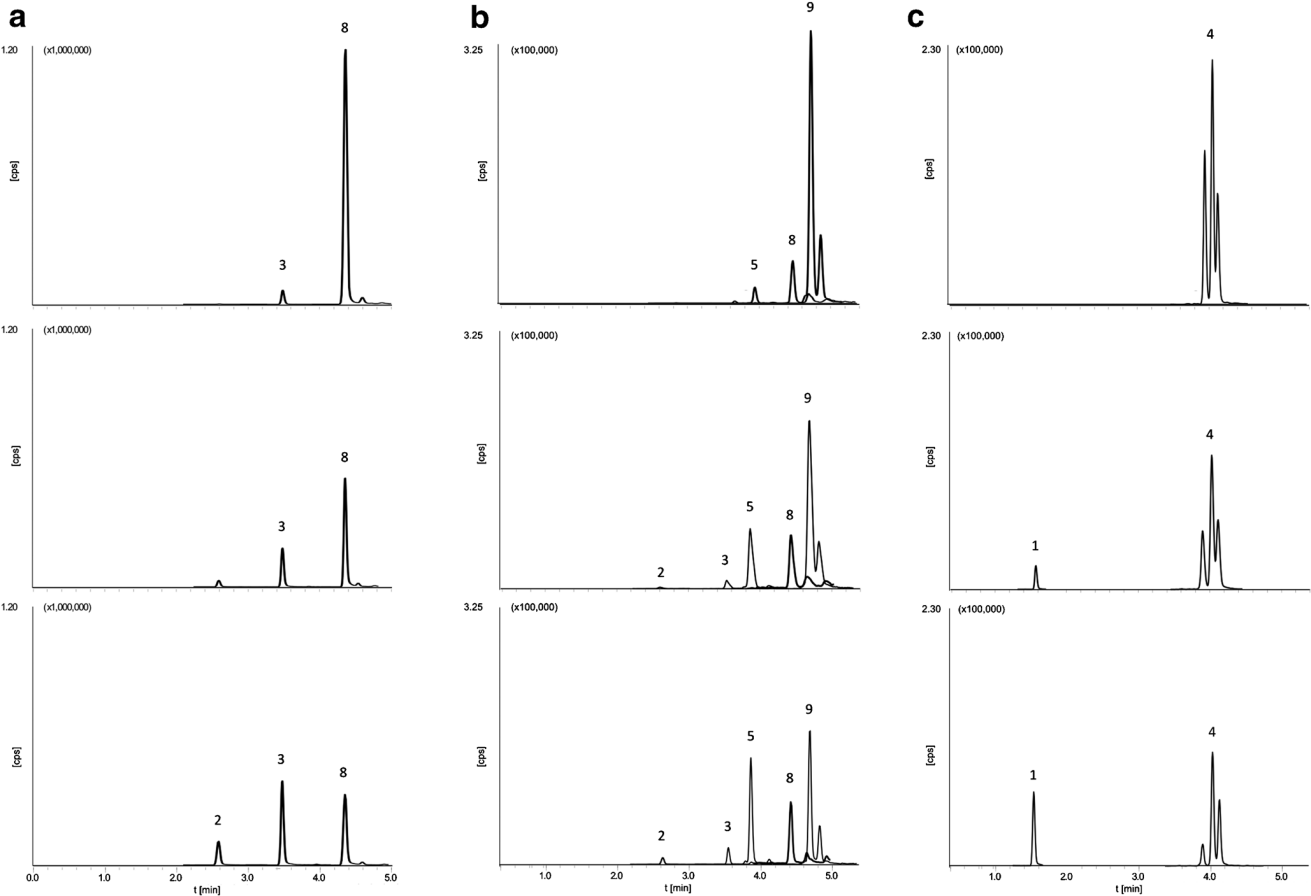
Effects of the time and temperature on the stability of **a** BADGE, **b** BADGE·HCl, and **c** BFDGE. Analytes: (1) BFDGE·2H_2_O, (2) BADGE·2H_2_O, (3) BADGE·H_2_O, (4) BFDGE, (5) BADGE·H_2_O·HCl, (8) BADGE, and (9) BADGE·HCl

Because the derivatives were present in the initial compound mixture, it was necessary to exclude their impact on the obtained results. We have shown that the selected compounds can be transformed into their derivatives (BADGE into BADGE·H_2_O and BADGE·2H_2_O; BADGE·HCl into BADGE·H_2_O·HCl, BADGE, BADGE·H_2_O, and BADGE·2H_2_O; and BFDGE into BFDGE·2H_2_O); thus, it was necessary to check their behavior in solutions containing only one compound. The specific compounds were chosen on the basis of conducted research, and experiments were repeated under conditions that had the most significant influence on the transformation. Only solutions with methanol contents of 40.0%, 20.0%, and 0.1% and two temperatures (4 and 20 °C) were selected because they had an observable impact on the conversion rate. To confirm the conversion of BADGE, BADGE·HCl, and BFDGE under specific conditions, single sets of these solutions were prepared in the same manner as described. Briefly, a working solution of each analyte was diluted with an MeOH/H_2_O mixture (10.0 mL each). The concentration of each analyte was 100.0 ng/mL. The BADGE, BADGE·HCl, and BFDGE underwent transformations to their derivatives. A comparison of the chromatograms of BADGE, BADGE·HCl, and BFDGE studied after the 1st, 7th, and 14th days is shown in Fig. 4.

Details of the impact of the time, storage temperature, and water content on the stability of investigated analytes are summarized in ESM Fig. S2. On the basis of the data, we concluded that the storage temperature had a significant effect on the transformation rate. Moreover, the stability of these compounds decreased with increasing solution water content. The most significant concentration changes were detected for samples with the highest water content and stored at 20 °C. Interestingly, on the basis of the results obtained, the majority of cases transformation processes occurred after only 1 day.

## Conclusions

In this study, we aimed to determine the effect of the composition and the storage conditions of model solutions on the stability of the tested analytes. The obtained results indicate that the stability of the analytes decreased with increasing solution water content. In addition, the use of low storage temperatures (4 and − 20 °C) only slightly reduced the concentrations of the monitored analytes. Reductions in the BADGE and BFDGE concentrations were observed, along with increases in the concentrations of BADGE·H_2_O, BADGE·2H_2_O, BADGE·H_2_O·HCl, and BFDGE·2H_2_O. This indicates that the presence of water caused the transformation of BADGE and BFDGE into their respective derivatives. Minor changes in the compound concentrations were observed in samples with the highest content of the organic solvent (≥ 80.0% MeOH). Consequently, it can be assumed that under these conditions, these compounds are stable, and their degradation does not influence the results significantly.

The obtained data indicate that the transformations of BADGE and BFDGE occur not only in contact with packaging during processing but also in the laboratory under controlled conditions. Furthermore, in solutions with a high water content, three-ring NOGE and BADGE·HCl were deposited on the walls of the vial. On the basis of our results, the best quality analytical results can be obtained by limiting the water content of the solutions. It is advisable to prepare the standard solutions just before commencing any further instrumental or biological studies. Moreover, in this study, a rapid and sensitive HPLC–MS/MS method for the simultaneous analysis of analogs of BADGEs and BFDGEs was developed. Highly efficient separation (in less than 6.0 min) was obtained by using a C_8_ core-shell column. To date, no information concerning whether and to what extent the solution composition and the storage conditions affect the stability of the tested compounds has been presented. Hence, this study is a valuable source of information and provides guidance for improving the quality of analysis of BADGE, BFDGE, and their derivatives.

Global trends in the analytical chemistry of food contact materials are currently being shaped and developed [13, 34]. Clearly, it is vital that research into combining short- and long-term simulations, as well as migration studies, using unified and validated interlaboratory protocols and methodologies, both instrumental and biological, is carried out. This will allow comprehensive and holistic information about the changes in food properties resulting from the use of packaging materials and storage to be provided.

## Electronic supplementary material

ESM 1 (PDF 1351 kb)
