# Supplementary material for: Stabilities of bisphenol A diglycidyl ether, bisphenol F diglycidyl ether, and their derivatives under controlled conditions analyzed using liquid chromatography coupled with tandem mass spectrometry
Source: Anal Bioanal Chem. 2019 Jul 19;411(24):6387–98. doi: 10.1007/s00216-019-02016-5 (PMC6718377; doi:10.1007/s00216-019-02016-5)
Supplement: Supplementary file 1 — (PDF 1351 kb) [file 216_2019_2016_MOESM1_ESM.pdf]

Analytical and Bioanalytical Chemistry

Electronic Supplementary Material

Stabilities of bisphenol A diglycidyl ether, bisphenol F diglycidyl ether, and their derivatives under controlled conditions analyzed using liquid chromatography coupled with tandem mass spectrometry

Natalia Szczepańska, Paweł Kubica, Błażej Kudłak, Jacek Namieśnik, Andrzej Wasik

a) BADGE HCl

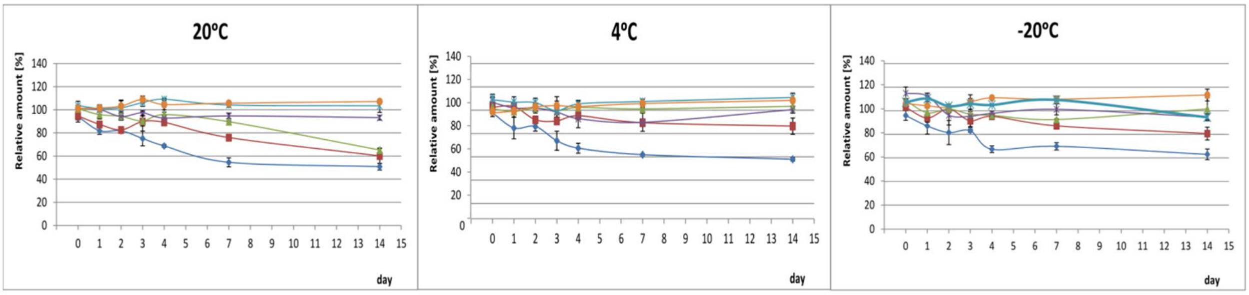

b) BFDGE $2H_2O$

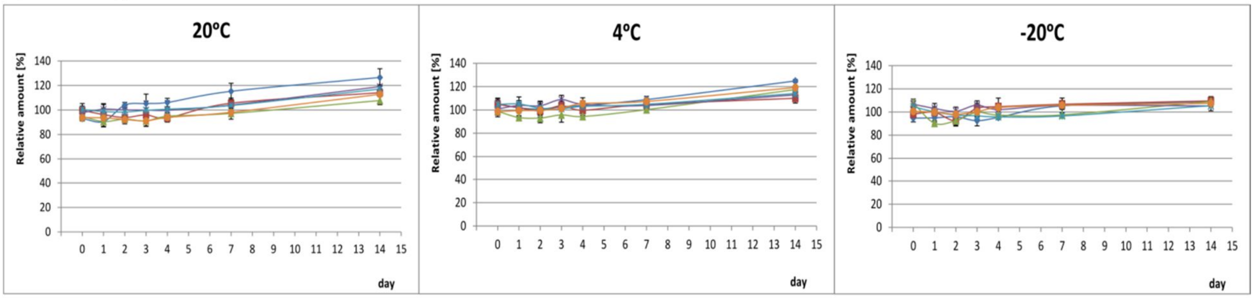

c) BADGE $2H_2O$

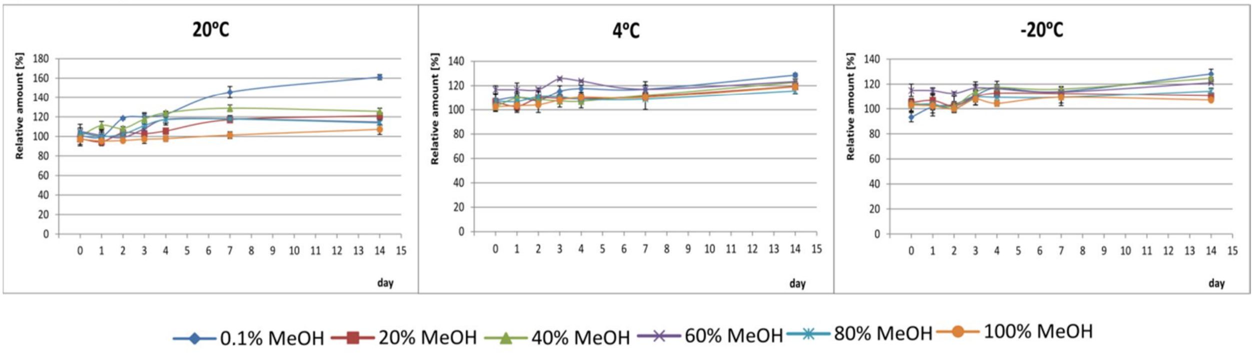

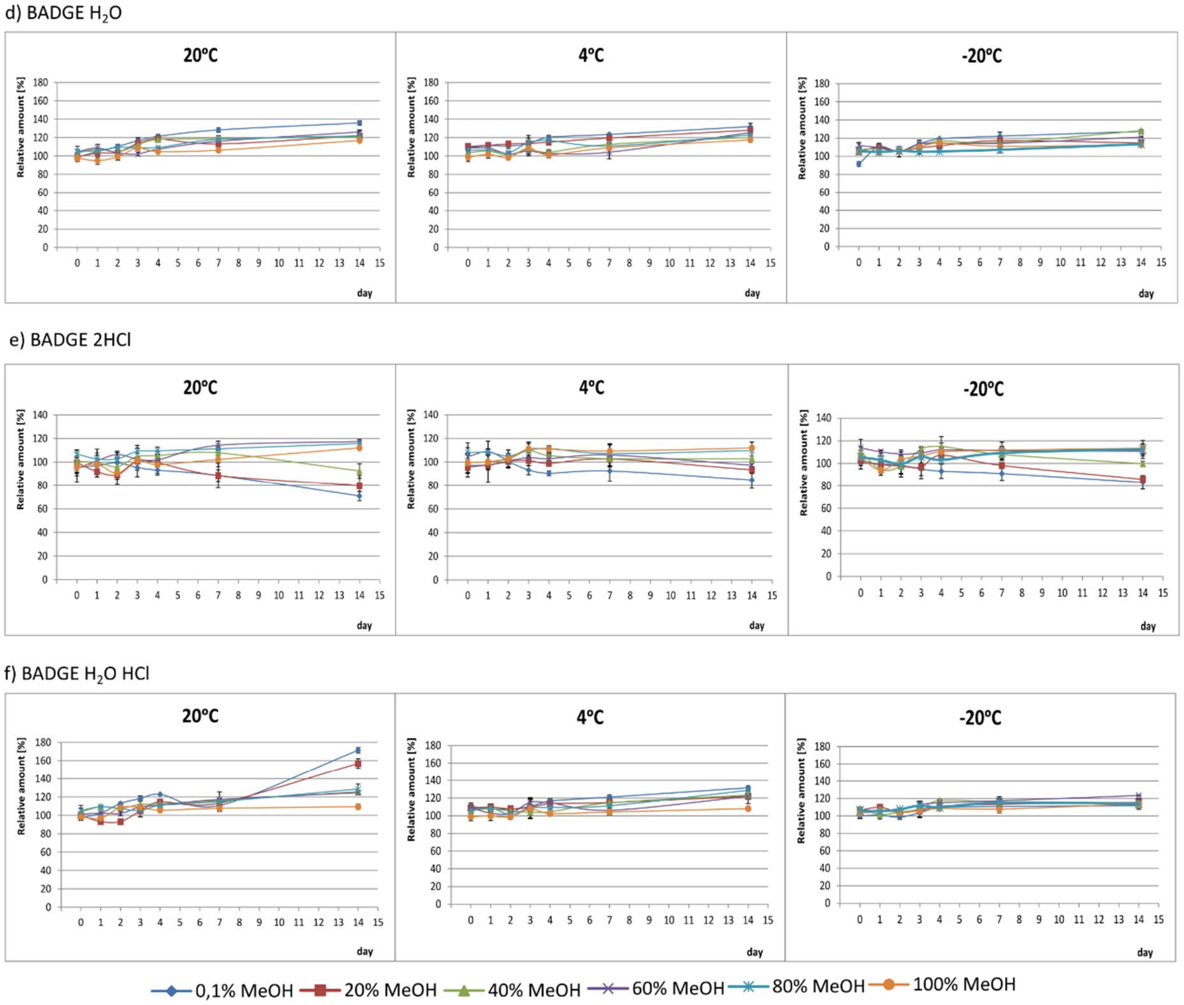

Fig. S1 Results of influence of the time and temperature on the stability of a) BADGE·HCl, b) BFDGE·2H_2O, c) BADGE·2H_2O, d) BADGE·H_2O, e) BADGE·HCl, f) BADGE·H_2O·HCl

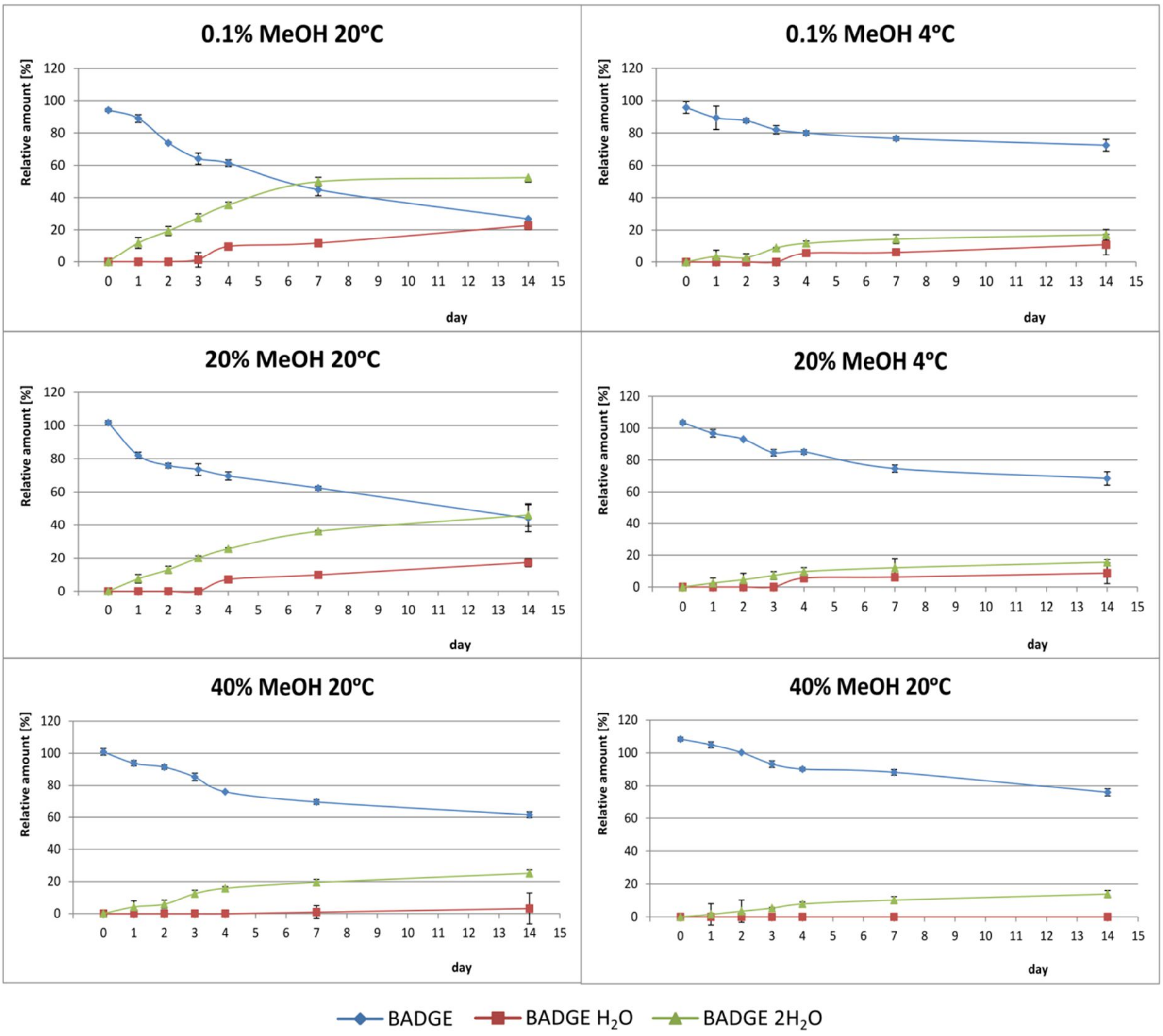

Fig. S2 Results of influence of the time and temperature on the stability of BADGE

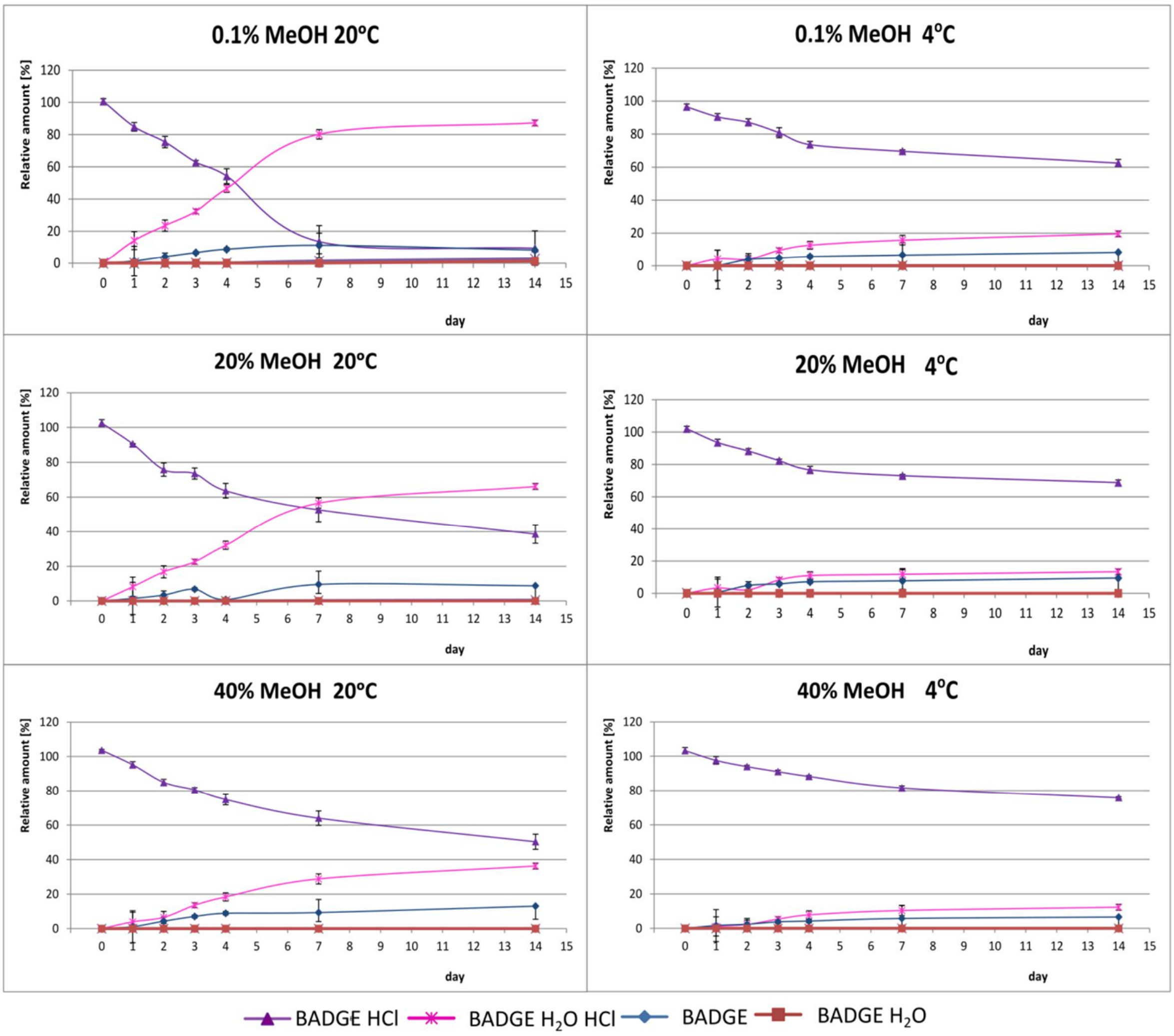

Fig. S3 Results of influence of the time and temperature on the stability of BADGE·HCl

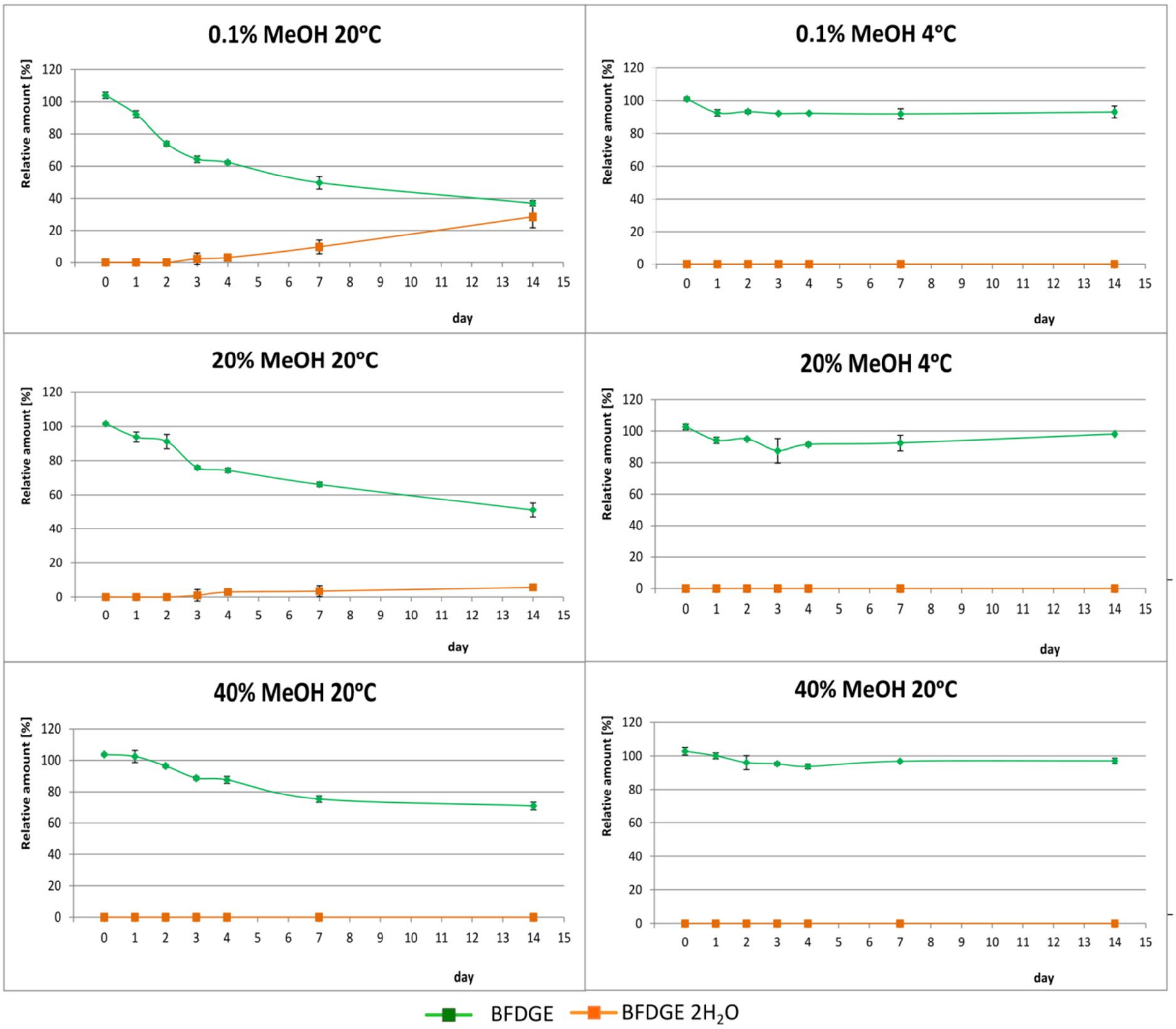

Fig. S4 Results of influence of the time and temperature on the stability of BFDGE
